# Factors associated with access to health services and quality of life in knee osteoarthritis patients: a multilevel cross-sectional study

**DOI:** 10.1186/s12913-019-4441-2

**Published:** 2019-10-11

**Authors:** Siriwan Choojaturo, Siriorn Sindhu, Ketsarin Utriyaprasit, Chukiat Viwatwongkasem

**Affiliations:** 1 Department of Surgical Nursing, Faculty of Nursing, Mahidol University, Bangkok, 10700 Thailand; 2 Department of Biostatistics, Faculty of Public Health, Mahidol University, Bangkok, 10400 Thailand; 3 Mahidol University, Faculty of Nursing, 2 Wang Lang Road, Siriraj, Bangkoknoi, Bangkok, 10700 Thailand

**Keywords:** Health service system, Osteoarthritis, Chronic disease management, Quality of life, Access, Self-management

## Abstract

**Background:**

The main purpose of health service systems is to improve patients’ quality of life (QoL) and to ensure equitable access to health services. However, in reality, nearly half of knee osteoarthritis (OA) patients present to the health system do not have access to health services, and their QoL remains poor. These circumstances raise important questions about what (if any) factors can improve health care accessibility and QoL for knee OA patients.

**Methods:**

A multicenter, cross-sectional survey was performed with 618 knee OA patients who received care at 16 hospitals in Thailand. Structural equation modeling (SEM) was conducted to investigate the association of health service factors and patient factors with access to health services and QoL.

**Results:**

The QoL of knee OA patients was very poor (mean score = 33.8). Only 2.1% of the knee OA patients found it easy to obtain medical care when needed. Approximately 39.4% of them were able to access appropriate interventions before being referred for knee replacement. More than 85% of orthopedic health services had implemented chronic disease management (CDM) policy into practice. However, the implementation was basic, with an average score of 5.9. SEM showed that QoL was determined by both health system factors (β = .10, *p* = .01) and patient factors (β = .29, *p* = .00 for self-management and β = −.49, p = .00 for disease factors). Access to health services was determined by self-management (β = .10, p = .01), but it was not significantly associated with QoL (β = .00, *p* = 1.0).

**Conclusions:**

This study provides compelling information about self-management, access to health services and QoL from the individual and health service system perspectives. Furthermore, it identifies a need to develop health services that are better attuned to the patient’s background, such as socioeconomic status, disease severity, and self-management skills.

## Background

In large epidemiology studies, osteoarthritis (OA) has been reported as a major common cause of extremely poor quality of life (QoL) for hundreds of millions of adults of all ages worldwide [1]. The QoL of knee OA patients was found to be almost 50% lower than that of non-OA patients [2], and patients with end-stage OA reported a health state equal to or worse than that of patients with other chronic diseases [3]. OA has all the hallmarks of a harmful condition. It has been found to have the fourth greatest impact on the overall health of the world population in terms of both death and disability [4], highlighting the need for health system reforms.

In 2000, a worldwide campaign called the “Bone and Joint Decade” was launched to address this burden [5, 6]. Additionally, the Institute of Medicine (IOM) has advocated the Chronic Disease Management (CDM) policy to redesign the health service system at all levels [7, 8]. Since then, many countries, including Thailand, have established and implemented health policy, strategy, and planning at a national or institutional level. Subsequently, OA guidelines have been developed and implemented to provide a standard of care and assist physicians worldwide in tailoring OA management [9]. However, in reality, nearly half of knee OA patients in the health system do not have access to optimal health services, and their QoL remains immensely poor [10, 11], even in developed countries where health resources are plentiful.

Traditionally, physicians have had the authority to tell patients what to do and have expected patients to follow their orders. They have also tended to use management options such as medication and surgery for quick solutions, with a great focus on pain management [12, 13]. However, the actual provision of physician services remains limited. Safety, long waiting times, and health financing have been identified as problems [14]. Consequently, knee OA patients have tried to manage their care themselves, and they needed appropriate information and practical skills [15, 16]. They desire to live well with their condition [17]. Therefore, responsibility for the management of OA overlaps among patients, providers, and health systems.

The prevalence of knee OA in developing countries, including Thailand, is generally similar to that seen in high-income countries (HIC) [18]. In addition, the feasibility of implementing a full multidisciplinary care team in Thailand given the restrictions of a limited health workforce and funding is low. A rational prediction is that the impact of the disease may be extreme because of the low general standard of living. However, the situation is more complex than this. The body of evidence substantiates that access to health services and QoL are complex constructs that are correlated with health service system factors and patient factors. However, a dearth of research on the topic is available. Furthermore, the effect of interplay of patient factors and health service system factors on access to health services and QoL has rarely been explored. To address this evidence gap, research is needed to determine whether certain factors can improve access to health services and QoL in knee OA patients.

## Methods

### Study design and setting

A multilevel, hospital-based survey was conducted at 16 hospitals in Thailand from June 2015 to June 2016 to determine how health service system factors and patient factors are associated with access to health services and QoL in patients who have been diagnosed with knee OA. A structural equation modeling (SEM) analysis was used to test the effect of the exogenous (health service system factors and patient factors) construct variables on the endogenous (access to health services and QoL) construct variables.

### Study participants

The sample in this study consisted of 2 groups. One comprised 16 groups of health care providers from 16 hospitals. The other comprised 618 patients diagnosed with knee OA based on radiographic evidence and/or American College of Rheumatology clinical criteria.

#### Sampling

The researcher employed a proportionate stratified random sampling technique by service provider characteristics to obtain research participants.

In the first stage, 8 hospitals offering tertiary health services were selected by random sampling from a list of 56 hospitals in 34 provinces that followed the service plan of the Ministry of Public Health, Thailand [19]. The hospitals were eligible to participate if they were regional or universal hospitals, had orthopedists with specialty or subspecialty training in the areas of hip-knee treatment, and provided comprehensive, multidisciplinary care.

In the second stage, 8 hospitals offering primary health services were selected by random sampling from the list of hospitals within the same province as 8 hospitals offering tertiary health services. All the hospitals were eligible to participate if they were district or community hospitals and had general doctors and health care teams to provide generalist medical care and referred patients in need of more advanced conditions to tertiary health services. Consequently, 16 groups of healthcare providers who had relevant knowledge or expertise in orthopedic care and who were full-time regular employees were purposively selected from each hospital.

Finally, a total of 618 individual knee OA patients were selected who met the following eligibility criteria: (1) age 18 years and older; (2) a diagnosis of knee OA made 12 months or more before data collection began; and (3) kept at least 80% of appointments within the 12 months before their participation in the study. The patients were selected by random sampling from the total number of knee OA patients within each hospital. Individuals were excluded if they had cognitive dysfunction or demonstrated an unstable condition during the study (i.e., respiratory failure or unstable angina).

## Outcome measures

### Quality of life

The Osteoarthritis Knee and Hip Health-related Quality of Life (OAKHQOL) is a disease-specific QoL questionnaire for patients with knee and hip OA [20]. It is self-administered and consists of 43 items in five dimensions. The dimension scores are standardized from 0 (worst QoL) to 100 (best QoL). This QoL instrument has been used in several clinical and epidemiological studies [21]. The OAKHQOL has been shown to possess internal consistency, with alpha coefficients ranging from 0.80–0.96, and a good test-retest result, with interclass correlation coefficients ranging from 0.73–0.87. The construct and criterion validity have also been examined [22].

### Chronic disease management

The Assessment of Chronic Illness Care (ACIC) is an international questionnaire used to evaluate how well a practice team or organization implements chronic disease management (CDM) policy in practice [23]. The ACIC questionnaire consists of 32 items grouped into seven components. The component scores are standardized from 0 (no implementation) to 11 (full implementation). The ACIC has been shown to possess internal consistency, with alpha coefficients ranging from 0.85–0.97, and good test-retest results, with interclass correlation coefficients ranging from 0.87–1.00. The construct and criterion validity have also been examined [24].

### Access to health services

The Osteoarthritis Quality Indicators (OAQIs) is a specific quality indicator used to measure whether standards of care are provided for patients with knee and hip OA based on medical records [25]. The OAQIs consist of 15 items that are broadly applicable to current international guidelines for the assessment of nonpharmacological and pharmacological management of OA. Each item is answered with one of two checklist options (do, do not do). The number of indicators passed (items answered “do”) is divided by the number of eligible medical records and then multiplied by 100. The results are reported as a QI pass rate ranging from 0 to 100, with higher pass rate scores indicating greater access.

### Self-management (SM)

The Osteoarthritis Self-Management Screening (OASMS) is a self-administered questionnaire used to measure self-management skills; it was developed by researchers based on a literature review and the Perceived Medical Condition Self-Management Scales for patients with chronic disease [26]. The OASMS consists of 26 items in 4 dimensions: (1) perceived need; (2) self-efficacy; (3) knowledge of the skill; (4) and adherence to recommendations. Dimension scores are standardized from 0 (worst SM) to 100 (best SM). The internal consistency was tested, and alpha coefficients ranged from 0.82–0.93; a good test-retest result was determined, with interclass correlation coefficients ranging from 0.68–0.92. The construct and criterion validity have also been examined.

### Data collection

To collect data on patient factors and QoL, the 618 eligible patients were invited to complete a sociodemographic questionnaire, a clinical profile questionnaire, and the OAKHQOL questionnaire. To gather data on available hospital-based orthopedic services and the practicality of CDM implementation, the 16 groups of health care providers were invited to answer a hospital-based profile questionnaire and the ACIC.

Data regarding access to health services were extracted from the electronic and paper records of 618 patients, and access rates were measured in terms of 15 OAQIs.

### Ethics

The study received ethical approval through the Mahidol University Institutional Review Board (Ref. no. IRB-NS-2015/22.0303), which determined that the study posed no risks to participants. Informed consent was obtained from all individual participants included in the study.

Data collection was conducted in compliance with the Good Clinical Practices protocol and the Declaration of Helsinki. The participants received written and oral information about the study, and they provided their written informed consent prior to the baseline data collection.

## Results

In total, 618 knee OA patients were included in the analyses. The response rate was 100%. The mean age was 64.7 years (SD 8.6). Only 32.3% had completed secondary school. Most (77.9%) were employed, and 77.7% had a low income. More than two-thirds (68.3%) of the participants had been diagnosed in the severe stage. The mean QoL was 33.8 (SD 12.7), while the mean SM was 35.1 (SD 5.3) (Table 1).

**Table 1:** Sociodemographic and clinical characteristics of the study participants (*N* = 618)

Sociodemographic characteristics		N	%
Age	Mean = 64.7 (SD. = 8.6)		
Education level	Primary	418	67.6
	Secondary	90	14.6
	Tertiary	110	17.8
Occupation status	Unemployed	138	22.3
	Employed	480	77.7
Income status	None: lower than minimum wage	490	79.3
	Minimum wage to average income	87	14.1
	High income	41	6.6
Stage of disease, as diagnosed by doctor	Mild	63	10.2
	Moderate	133	21.5
	Severe	422	68.3
Quality of life	Mean = 33.8 SD (12.7)		
Self-management	Mean = 35.1 SD (5.3)		

The access rate percentage was 54.2 (SD 14.1). Nonsteroidal anti-inflammatory drugs (NSAIDs) and/or other types of analgesics were prescribed to all knee OA patients. Only 39.4% of knee OA patients could access appropriate interventions before being referred for total knee replacement (Table 2).

**Table 2:** Access to health services for the study participants (*N* = 618)

Indicator	Numerator/denominator	Access rate (%)
Access rate; mean (SD)	54.2 (SD14.1)	
Nonpharmacological access rate		
Holistic assessment	353/618	57.0
Weight control	262/328	79.9
Exercise advice	260/618	42.1
Health education	252/618	40.8
Assistive devices	45/284	15.9
Pharmacological access rate		
Adequate acetaminophen use	331/618	53.6
NSAIDs and COX-2 as adjuvant analgesic drugs	223/618	37.7
NSAIDs and/or other types of analgesics	618/618	100
NSAIDs plus PPI	215/335	64.2
NSAID use with appropriate risk assessment	182/335	54.9
All applicable indicators before referral for TKR	112/284	39.4

*NSAIDs* = nonsteroidal anti-inflammatory drugs; *COX-2* = cyclooxygenase 2; *PPI* = proton-pump inhibitor; *TKR* = total joint replacement

There was a non-statistically significant difference in the mean of CDM policy implementation (all components) between the different levels of health services, with a total CDM policy implementation mean score of 5.9 (Table 3).

**Table 3:** CDM policy implementation categorized by level of health service (*N* = 16)

CDM policy implementation	Level of health service				*p*-value
	Primary *N* = 8		Tertiary *N* = 8		
	Mean	SD	Mean	SD	
Health care organization	6.5	3.1	7.4	1.6	.51
Community resource	5.2	2.7	5.5	3.7	.88
Self-management support	5.5	3.0	5.5	1.6	1.00
Decision support system	5.4	3.0	5.4	2.0	1.00
Delivery system design	6.0	3.1	6.0	1.3	.97
Clinical information system	6.0	3.3	6.1	1.2	.93
Total CDM policy implementation; mean (SD)	5.9 (SD 2.0)				

*p* = .05, CDM = chronic disease management

**Fig. 1:**
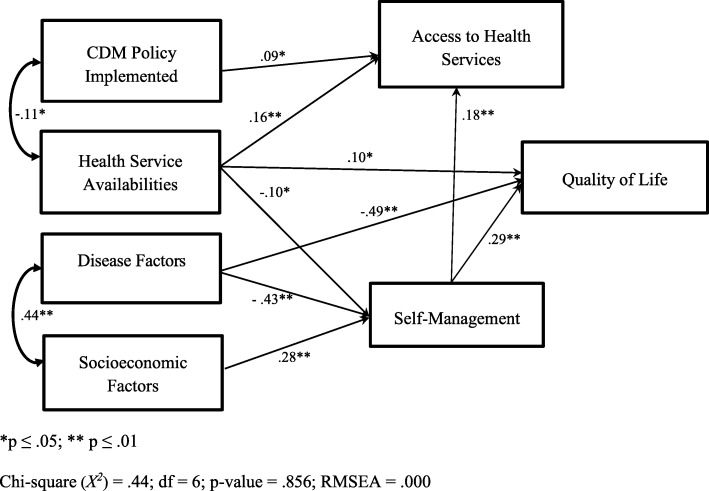
A modified model of the study

SEM showed that both health system factors (β **=** .10) and patient factors (β **=** .29 for SM and β **=** −.49 for disease factors) were directly or indirectly associated with QoL. However, the association between access to health services and QoL was not statistically significant. SM played an important mediating role and was correlated with access to health services (β **=** .13). This finding was robust across symptom severity and remained significant after other factors associated with QoL were adjusted for (Fig. 1).

## Discussion

This study demonstrated that the majority of knee OA patients were of early retirement age and had low socioeconomic status (SES). This finding could be attributed to economic factors as most elderly patients cannot afford to retire. It has been estimated that 80–90% of the population in low- and middle-income countries (LMIC) is involved in “heavy work”. There is evidence that knee OA patients with a lower SES have a poorer QoL [27]. However, this study found that SES factors affected QoL not directly but through SM. This finding is similar to findings in many recent studies that revealed that SES alone is not associated with QoL in knee OA patients [28–30].

The basic finding of this study was that worsening knee OA symptoms were related to the deterioration of QoL (β = −.49, *p* = .00). The QoL of knee OA patients appeared to be low. However, after symptom severity was adjusted for, the mean QoL scores in this study were closely linked to those found in the rest of the world [31–33]. This indicates that the QoL of knee OA patients is dependent on their disease severity rather than their country-specific context.

Additionally, this study found that knee OA patients with a higher SES might not have better access to health services. These data are inconsistent with those of previously published reports [34, 35]. One reason for this discrepancy is that this study was conducted in a country where national health care use tends to favor the poor, especially since the implementation of a universal coverage policy in 2011 and a free medical care program for destitute elderly people in 2009. These two programs help facilitate equal access to health services for all Thais by removing cost barriers [36].

Most importantly, this study found that knee OA patients who had better SM not only had better QoL (β = .29, *p* = .00) but also had better access to health services (β = .18, p = .00). This finding was robust across symptom severity levels and remained significant after many factors associated with QoL were adjusted for. Findings from qualitative studies have indicated that the majority of knee OA patients view individual responsibility as a key to maintaining health and living well with knee OA [13]. Conceivably, OA patients’ desire to manage the impact of their condition on their life facilitates SM. Patients in a study by Miller et al. [37] reported that they attempted to adapt their lifestyle to reduce knee joint symptoms. Some patients took the initiative to start medication and nonpharmacological treatment on their own. Most of these patients sought information to gain control over their condition using a variety of sources, such as multimedia platforms and peers [38]. Therefore, they also had to access appropriate information to increase their knowledge how to self-manage their problem to improve QoL.

Interestingly, SM was strongly linked to patients’ socioeconomic (SES) factors (β = .28, *p* = .00) and disease factors (β =. -43, p = .00). Thus, QoL improved or worsened the effects of SES and disease factors on patients’ ability to manage their own disease. The mechanisms for the association between SES and SM remain unclear. Indirect evidence suggests that SES plays a pivotal role in the lives of citizens [39]. It is essential that patients with low SES receive sufficient knowledge to empower them to actively manage their health.

Unsurprisingly, knee OA patients attempt SM when their symptom severity increases. This finding contrasts with the findings of previous research showing that COPD patients who reported a higher symptom burden also reported lower SM [40]. There is a misbelief that knee OA is just part of the aging process. Knee OA patients might simply be told that there is nothing to be done except knee arthroplasty [41]. Although health care professionals state that SM is useful in improving patients’ health outcomes, it is often completely under recognized and omitted from patient education [42, 43]. Thus, knee OA patients are left underprepared for SM when their symptom severity is mild. Furthermore, most health care providers have focused on SM-related support rather than on patient SM. However, a recent review reported that SM support alone likely has little to no effect on improving knee OA patients’ health outcomes [44].

Knee OA patients have expressed disappointment that the public health care system does little to help them [45]. Given this reality, to improve patients’ SM, a better understanding of how to deliver health services and align evidence with policy and practice is required. We must also reshape our mindsets and imagine ourselves as patients, particularly those with SES disadvantages. Increasing awareness among the public and clinicians regarding the multifaceted impact of OA on the lives of a large segment of the population may increase attention to the problem and elicit more proactive management by both patients and physicians.

The present study has several limitations. First, it was conducted in government hospital settings with specific recruitment requirements. Thus, it cannot be extrapolated to other settings (private hospitals, home health care, populations with other ethnic backgrounds, etc.). The data were collected in a single country. Therefore, the results of this study may not always be extrapolated to countries with different health care policies. This was not a placebo-controlled trial; thus, the extent to which QoL improvements may have been related to placebo effects cannot be estimated. Other limitations of the study are that the access rates may not reflect the care quality, as perceived by the patients, and health care providers may interpret the quality differently from their patients. However, self-reports might represent a weakness because there is potential for recall bias, which might have led to over- or underestimated access rates in the study.

A key strength of this study of knee OA patients is that it provides powerful information about access to health services and QoL from the individual and health system perspectives. One of the first steps toward improving QoL in adults with OA is to develop a better understanding of the factors that coexist with and influence QoL. Most studies focus on single variables concerned with individual factors (e.g., gender, education level, income, career and severity of disease), while QoL involves multiple factors. This study strongly supports the notion that SM is independently correlated with improved QoL. Therefore, understanding patient SM is important to identifying other factors for best practice and potential strategies to create more patient-centered health services for OA care.

## Conclusions

This study highlights the strengths of existing OA clinical practices and the opportunity to create health service systems that positively influence future care. Health service system factors and patient factors all appear to influence QoL in knee OA patients. Most importantly, the effective management of OA care begins and ends with the patients. Moreover, there is a need to target research efforts toward specific interventions to improve patient SM, which remains a key patient factor.

## Data Availability

Data are available upon reasonable request from the corresponding author. The raw data that support the finding of this study are available in the [figshare] repository with the identifier [DOI: 10.6084/m9.figshare.7784792].

## Abbreviations

CDM: Chronic disease management
OA: Osteoarthritis
QoL: Quality of life
SES: Socioeconomic status
SM: Self-management
